# Last Conscious Thoughts

**Published:** 1963-10

**Authors:** G. De M. Rudolf


### LAST CONSCIOUS THOUGHTS

BY

G. De M. RUDOLF, M.R.C.P., D.P.H., D.P.M.

The last conscious thoughts before loss of consciousness have been of interest for many years. Some people have been reported as having said that they had had a glimpse of an after-life, and had regretted returning to this one; others have recalled, so novelists write, a misspent youth, or events they wish had not taken place.

During an investigation into retrograde amnesia (Rudolf, 1947, 1948) enquiries were made with regard to the last memories before loss of consciousness, and in some of the patients questioned it was found that there was no period of amnesia. These patients therefore provided true last thoughts. Whether death takes place, or recovery occurs from unconsciousness, is a matter of chance and organic damage. Consequently the last thoughts before loss of consciousness as recalled by a person who has recovered and who recalls the injury sustained may be reliably supposed to be the same as they would have been if he had died without recovering consciousness.

If retrograde amnesia has occurred, however brief its duration, the last thoughts before injury may have been repressed. This repression is likely to be caused by the fear of injury or death. If conscious or unconscious fear is present, death would not be welcomed and an after-life would not be considered as pleasant. It is therefore possible that when death and its sequelae are feared, a person may have no last thoughts available for recollection. If on the other hand the individual believed that he was passing to an after-life of happiness, or returning to a state of non-existence, the event of death would not be unwelcome, and repression affecting the events leading up to death would then be unlikely to occur.

The present paper deals with 34 men who had suffered from unconsciousness but who had no retrograde amnesia—that is, they recollected the actual event, such as a blow, that produced unconsciousness. Twenty-five of these men were in action at the time of the incidents, and so were in the presence of danger and death. Of the total number of 37 periods of unconsciousness amongst the 34 men, 9 were accompanied by thoughts that occurred after the injury and before loss of consciousness. In one incdent the thoughts could have been memories of an actual event; in another incident unconsciousness could have commenced while a man was in the air, as he recalled rising but not falling. Thoughts recorded, which appeared not to be those of real events only, were as follows:

**Case 2.** Recalled bullet striking forehead, then felt as if rising in the air and falling.

**Case 4.** Recalled sound of explosion, and of going up in the air with head expanded.

**Case 23.** Electric shock; recalled smell of burning flesh and of being drawn-in to the apparatus, but in fact was thrown away from it to a distance of 5 yards. Skin was severely burned.

**Case 26.** Recalled explosion followed by a sensation of floating down, and finding himself lying across the threshold of a door.

**Case 32.** Recalled being hit on the head, followed by a feeling of falling down and down, as if from a cliff, and never reaching the bottom.

**Case 33.** On one occasion when wounded by shrapnel felt as if struck on the jaw (but was struck in left parietal region). On a subsequent occasion, recalled an explosion and felt he was being torn apart.

None of these thoughts dealt with past regrets or an after-life, although one of them, the sensation of falling down and down, might have been pleasant. Although these suddenly precipitated thoughts gave no vision of an after-life, this fact is no proof that such visions do not occur. The enquiries were made a considerable time after the injuries were sustained, and falsification of memory could have occurred. Somerville (1931) gives an instance of a cyclist believing for at least 17 years that he (Somerville) had knocked him down, as he was the first person the cyclist saw on regaining consciousness, whereas some other person had actually caused the incident.

Whether or not the thoughts described by these men had occurred, the patients believed that they had. This is also the situation in the case of persons who have been near death and yet have recovered, and who report that they have had a glimpse of an after-life. If a person asserts that he has seen a glimpse of an after-life, it is impossible to show that he has not. In the pursuit of truth, a belief that he has not is of the same value as a belief that he has.

### REFERENCES

Rudolf, G. de M. (1947). *Jl. mental Sci.*, **93**, 342.
Rudolf, G. de M. (1948). *Jl. mental Sci.*, **94**, 641.
Somerville, C. W. (1931). *Edin. Med. Jl.*, Trans Med. Chi. Soc., **38**, 121.

